# Lin28B promotes Müller glial cell de-differentiation and proliferation in the regenerative rat retinas

**DOI:** 10.18632/oncotarget.10343

**Published:** 2016-06-30

**Authors:** Zui Tao, Chen Zhao, Qian Jian, Mark Gillies, Haiwei Xu, Zheng Qin Yin

**Affiliations:** ^1^ Southwest Hospital/Southwest Eye Hospital, Third Military Medical University, Chongqing, 400038, China; ^2^ Key Laboratory of Visual Damage and Regeneration and Restoration of Chongqing, Chongqing, 400038, China; ^3^ Save Sight Institute, South Block, Sydney Eye Hospital, Sydney, NSW, 2001, Australia

**Keywords:** Müller glia cell, de-differentiation, Lin28B, let-7

## Abstract

Retinal regeneration and repair are severely impeded in higher mammalian animals. Although Müller cells can be activated and show some characteristics of progenitor cells when injured or under pathological conditions, they quickly form gliosis scars. Unfortunately, the basic mechanisms that impede retinal regeneration remain unknown. We studied retinas from Royal College of Surgeon (RCS) rats and found that let-7 family molecules, let-7e and let-7i, were significantly overexpressed in Müller cells of degenerative retinas. It demonstrated that down-regulation of the RNA binding protein Lin28B was one of the key factors leading to the overexpression of let-7e and let-7i. Lin28B ectopic expression in the Müller cells suppressed overexpression of let-7e and let-7i, stimulated and mobilized Müller glia de-differentiation, proliferation, promoted neuronal commitment, and inhibited glial fate acquisition of de-differentiated Müller cells. ERG recordings revealed that the amplitudes of a-wave and b-wave were improved significantly after Lin28B was delivered into the subretinal space of RCS rats. In summary, down-regulation of Lin28B as well as up-regulation of let-7e and let-7i may be the main factors that impede Müller cell de-differentiation and proliferation in the retina of RCS rats.

## INTRODUCTION

In many human retinal degenerative diseases, such as retinitis pigmentosa (RP) and age-related macular degeneration (AMD), the degeneration of photoreceptors and retinal neurons is an irreversible cause of vision loss or blindness for which there is currently no effective treatment [1]. Stem cell-based cell therapy of retinal degenerative diseases holds great promises. On the other hand, stimulating and mobilizing endogenous stem/progenitor cells is an attractive therapeutic candidate for retinal regeneration [2, 3].

Müller cells, the principal retinal glial cell, are anatomically and functionally essential for retinal development and homeostasis [4]. Under pathological conditions, Müller cells in fish and amphibians do not lose their ability to de-differentiate into progenitor cells, re-enter the cell cycle, and undergo proliferation [5–8]. In higher vertebrates, Müller glia have been reported to possess the properties of progenitor cells and generate new neurons after injury [9–11], suggesting that Müller cells might be endogenous stem/progenitor cells of retinas. However, in higher vertebrates, the inherent de-differentiation response of Müller cells to trauma appears to be too weak to repair the injured retinas. Recent studies have conclusively demonstrated successful promotion of Müller cell-derived functional neuronal regeneration in mammalian retina following acute injury or initiation of chronic retinal diseases [12–14].

Müller cell morphology is important since the cells extend into the subretinal space which may protect the retina by clearing dead cells and promoting the healing response. In mammals, retinal injury originally causes Müller cells to be active, which may alter their morphology, ability to proliferate and hypertrophy [15, 16]. However, with the development of retinal degeneration, excessive Müller cell activation and proliferation eventually result in the formation of glial scar in the subretinal space [17, 18], which aggravate photoreceptor apoptosis and impede photoreceptor regeneration even after successful retinal reattachment surgery [19, 20]. Therefore, the response of Müller cells can be a double-edged sword for retinal regeneration. Further research is necessary to clarify the mechanisms related to Müller cell mobilization and stimulation of de-differentiation that causes them to differentiate into target cells while preventing de-differentiated Müller cells from acquiring a reactive glial phenotype.

Lin28 is a RNA binding protein. In vertebrates, it is expressed in the central nervous system (CNS) during early embryonic development and histogenesis [21, 22] and plays an important role in regulating neurogenesis in developing brains [23] and retinas [24]. Over-expression of Lin28 in early retinal progenitors usually preferentially generates neurons and inhibition of Lin28 expression in late retinal progenitors usually generates Müller cells. The expression of Lin28 was significantly increased in the retina of zebra fish after injury, which promoted Müller cells to proliferate and de-differentiate into retinal progenitors by negatively regulating the expression of microRNA let-7 [25, 26]. MicroRNA let-7 was involved in cell differentiation and inhibited tumorigenesis. The deficiency of let-7 can stimulate DNA replication and cell division [27], so it suggested that let-7, miR-125, and miR-9 were the key regulators of retinal progenitor cells in the early to late developmental stages [28, 29]. Experimental manipulation of the expression of these miRNAs changed the distribution of neuronal subtypes, while overexpression of Lin28 maintained the early progenitor state [30].

In this study, we used the Royal College of Surgeons rat (RCS-p+ rat), a model of retinitis pigmentosa, which has a recessive defect in the merTK gene [31] and exhibits defective retinal pigment epithelial phagocytosis as well as progressive photoreceptor degeneration [32, 33]. The rat model was used to explore whether ectopic expression of Lin28B can stimulate Müller cell de-differentiation, proliferation and promote them to trans-differentiate into retinal neurons by preventing them from acquiring a reactive glial phenotype.

## RESULTS

### Transient de-differentiation of Müller cells during retinal degeneration in RCS rats

**Figure 1:**
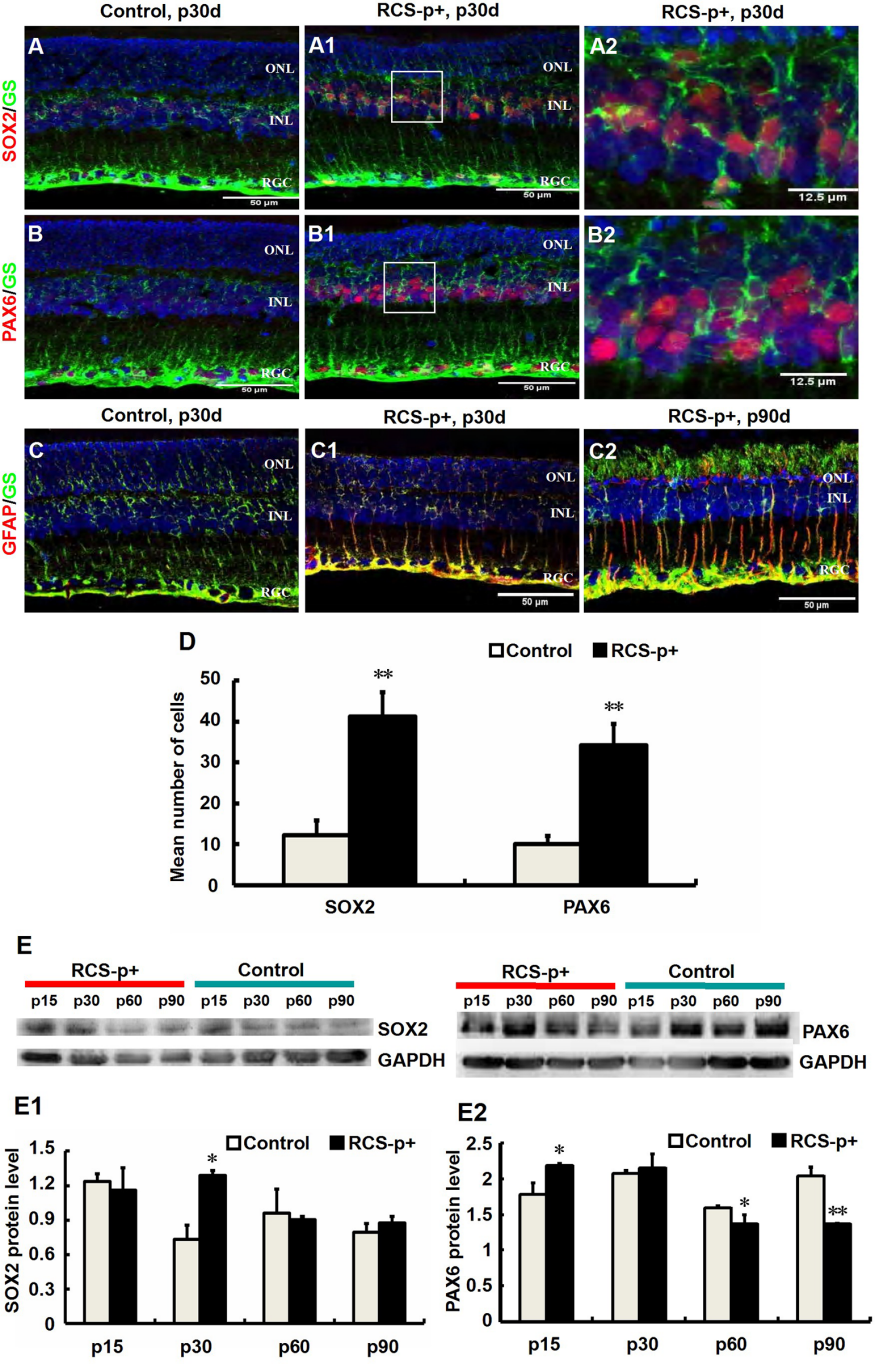
Transient Müller cells de-differentiation and final gliosis during retinal degeneration in RCS rats (**A**–**B2**) Double-label immunofluorescence staining against Müller cell marker GS (green) and progenitor markers SOX2 and PAX6 (red). (A2 and B2) are enlargements of the square parts of (A1 and B1). (**D**) Quantitative and statistical analysis of immunofluorescence staining. Compared with control rats' retinas, the levels of SOX2 and PAX6 in RCS-p+ rats' retinas were significantly increased during early degenerative stages (postnatal 30 days, p30d). (**E**–**E2**) Western blot analysis supported these results. (**C**–**C2**) Double-labeled immunofluorescence staining against GFAP (red) and GS (green). GFAP positive signals became detectable at p30 in RCS-p+ rat retinas, thereafter GFAP expression level gradually increased while no GFAP positive signals were observed in control rat retinas. Representative results are shown. Data are presented as the mean ± standard error from three replicates. *P < 0.05, **P < 0.01, Student's *t*-test.

We tested whether Müller cell de-differentiated during retinal degeneration in RCS-p+ rats and RCS-rdy+-p+ rats, a non-dystrophic, pigmented RCS rats as controls. Immunofluorescence double staining was performed, in which the antibody against glutamine synthetase (GS), a Müller cell marker, was used to double stain with antibodies against retinal stem/precursor markers, SOX2 and PAX6. We found that at an early stage of retinal degeneration, p30, in the inner nuclear layer (INL) of the retina, retinal stem/progenitor markers were expressed and co-localized with GS in Müller cell somas and their levels markedly increased in RCS-p+ rats compared with control rats (Figure 1A–1B2, 1D). These results were confirmed by western blotting. Expression of SOX2 and PAX6 increased at p15 and p30, and gradually decreased at middle and late stages of retinal degeneration, p60 and p90 (Figure 1E–1E2).

In contrast, we showed that the expression of glial fibrillary acidic protein (GFAP), a marker of Müller cell activation, appeared at p30 in the Müller cells of RCS-p+ rat retinas and increased with time and reached a peak at p90. The expression of GFAP in the Müller cells of control rats was not detected until p90 (Figure 1C–1C2) [34, 35]. These data suggested that retinal degeneration stimulates Müller cell de-differentiation into multipotent retinal progenitors at the early stages of degeneration in RCS-p+ rats, but these de-differentiated cells seem to become committed to a gliotic phenotype at the later stages of degeneration.

### De-differentiated Müller cells in degenerative rats' retinas only experienced a transient proliferative process

**Figure 2:**
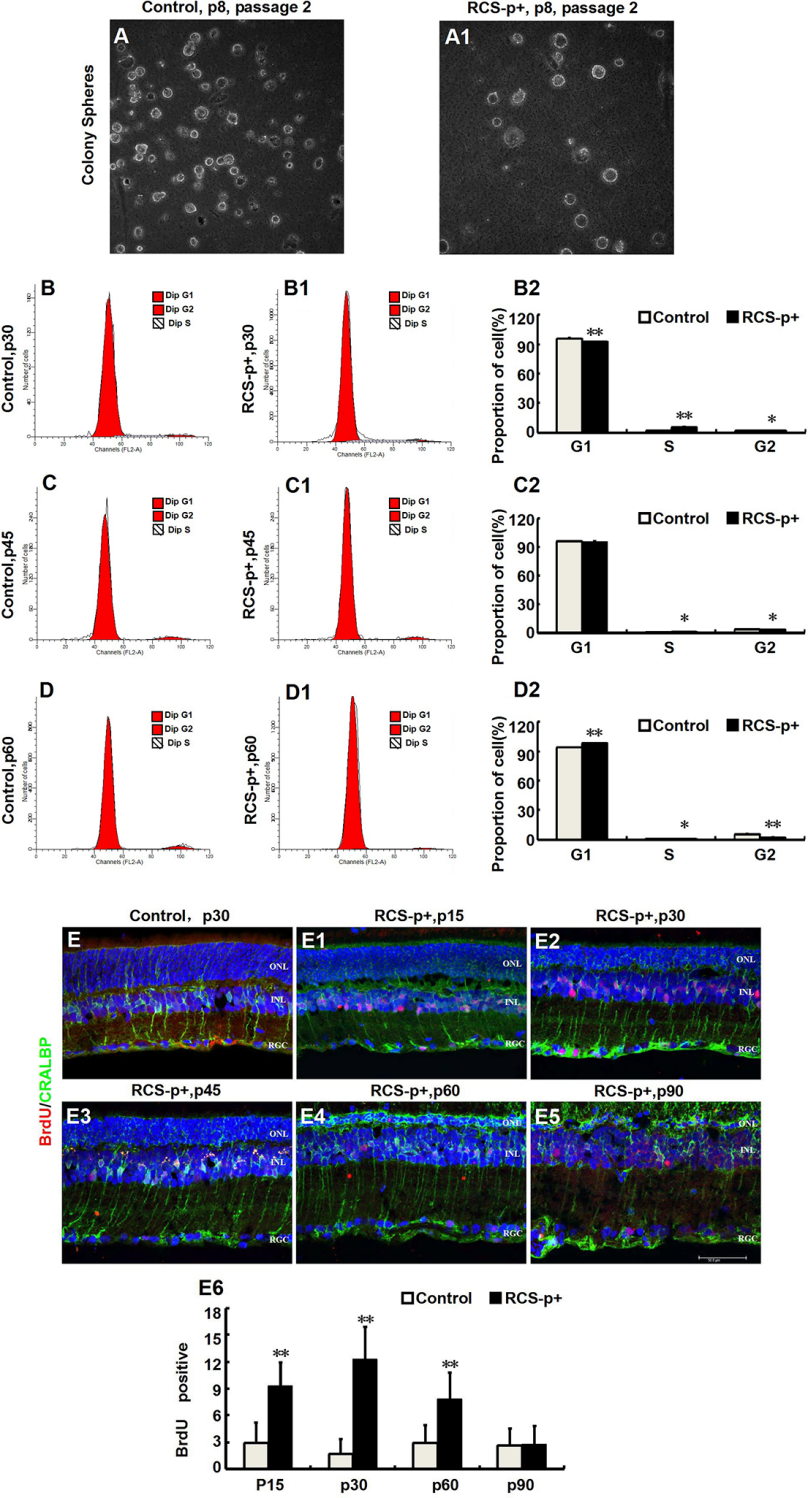
Transient Müller cells proliferation during retinal degeneration in RCS rats (**A**–**A1**) Müller cells isolated from retinas of RCS-p+ rats and controls at postnatal day 8 and cultured in medium with growth factors (EGF and bFGF). There was no significant difference between the two groups in the clone formation efficiency of retinal progenitor cells. (**B**–**D2**) DNA content analysis by flow cytometry after staining with propidium iodide. There was only a slight increase in the proportion of cells in the S phase in RCS-p+ rat retinas at p30 and p45 when compared with controls. Thereafter, the proportion of S phase cells in RCS-p+ retinas decreased. (**E**–**E6**) Double-label immunofluorescence staining against BrdU (red) and CRALBP (green) shows that Müller cells proliferation was observed during the early stage of retinal degeneration, with the number of double positive cells gradually reducing after p30. Representative results are shown. Data are presented as the mean ± standard error from three replicates. *P < 0.05, **P < 0.01, Student's *t*-test.

Stimulating de-differentiated cells to re-enter the cell cycle is a key process in replacing cells that have been destroyed by retinal degeneration. In order to study the self-renewal ability of de-differentiated Müller cells in RCS-p+ rat retinas, the Müller cells were dissociated into single cells and plated in stem cell medium at a clonal density (1 cell/μl). Although a few clonal spheres were observed in primary and first passage cultures, spheres were not observed in second passage cultures (Figure 2A and 2A1). To further verify the proliferative state of Müller cells in RCS-p+ and control rat retinas, cells were harvested at different time points for cell-cycle analysis by flow cytometry (FACS) after staining with propidium iodide. We found that there was only a slight increase of S phase cells at p30 and p45 in RCS-p+ rat retinas compared with controls and the number of S phase cells decreased at p60 (Figure 2B–2D2) in RCS-p+ rat retinas.

To confirm that Müller cells were dividing *in vivo*, the rats were injected intraperitoneally with BrdU (50 mg/kg) 2 h before sacrificing and the eyes were harvested. Using immunofluorescence double staining with the Müller glia specific marker CRALBP and the DNA replication marker BrdU, we found that a few Müller glia-derived progenitors started to express strong positive proliferative signals in the early stages of degeneration in dystrophic retinas compared with retinas from control rats (Figure 2E–2E5). At p15, the number of BrdU /CRALBP double labeled cells per field was significantly different 2.9 ± 2.3 vs. 9.3 ± 2.6 (*P* < 0.0001) for RCS-p+ and control rat retinas, respectively. The number of BrdU /CRALBP double labeled cells in RCS-p+ retinas reached a peak at p30 at which point there were significantly (*P* < 0.0001) more double positive cells in RCS-p+ retinas (12.3 ± 3.6 cells/per field) compared with controls (1.7 ± 1.6 cells/per field). This trend continued to p60 (2.9 ± 2.0 vs. 7.8 ± 3 cells/ per field, *P* = 0.001) and thereafter the number of double positive cells declined sharply in RCS-p+ retinas. There was no significant difference between the two groups at p90 (2.6 ± 1.9 for dystrophic rat retinas vs. 2.8 ± 2 cells/ per field for controls, *P* = 0.813) (Figure 2E6). Therefore, the level of BrdU labeled cells increased transiently, at p15 and p30, in dystrophic rat retinas compared to controls. Taken together, these data suggested that Müller cells proliferated in response to damage only at the early stages of retinal degeneration.

### Increased expression of let-7e and let-7i in the retinas of RCS rats

**Figure 3:**
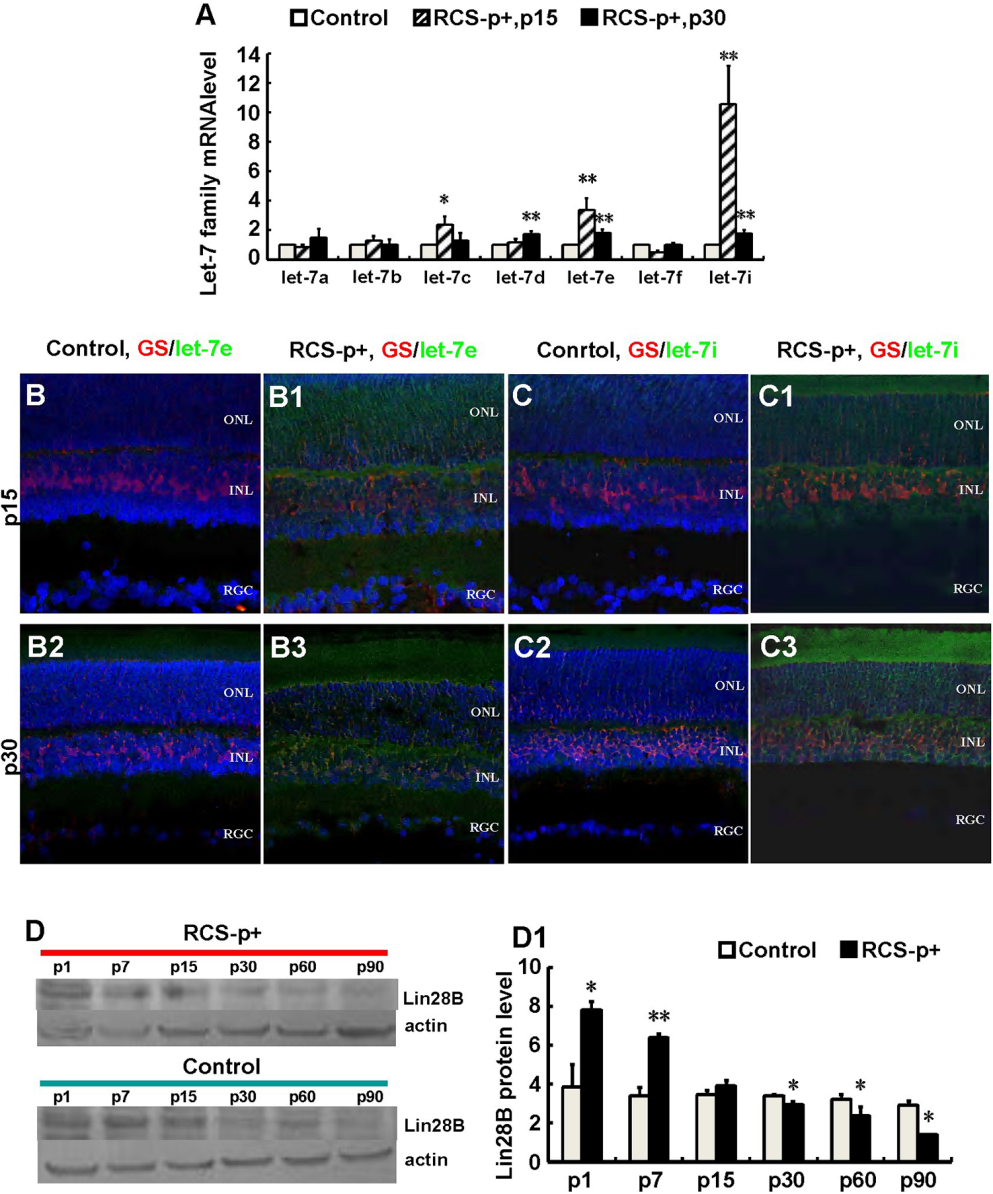
Upregulateion of let-7e and let-7i and downregulation of Lin28B in dystrophic rat retinas (**A**) Relative quantitative analysis showed that most members of the let-7 family, except let-7a and let-7f at p15, were upregulated at p15 and p30 in RCS-p+ rats' retina compared with controls. Among these members, let-7e and let-7i were upregulated most obviously. (**B**–**B3** and **C**–**C3**) Immunofluorescence simultaneously stained against GS (red) and *in situ* hybridization with LNA probes against let-7e or let-7i (green). The expression of let-7e and let-7i co-localized with GS in somas and processes of Müller cells. The intensities of these two molecular signals in RCS-p+ rat retinas were stronger than that of controls at early p15 and p30. (**D**–**D1**) Western blotting analysis showed that the expression of Lin28B protein only increased before retinal degeneration at p1 and p7, then was reduced after retinal degeneration at p15 in RCS-p+ rat retinas when compared with control rat retinas. Representative results are shown. Data are presented as the mean ± standard error from three replicates. *P < 0.05, **P < 0.01, Student's *t*-test.

In order to explore the underlying mechanisms for the inefficiency of Müller cells to re-enter the cell cycle during early stages of retinal degeneration, microRNA expression was quantified. The majority of the let-7 family was enriched and upregulated during the early stages of retinal degeneration, p15 and p30, in retina of RCS-p+ rats compared with controls. In RCS-p+ rats, let-7c, let-7e and let-7i, were upregulated 2.4 ± 0.6, 3.4 ± 0.8, and 10.6 ± 2.6 times at p15, respectively and upregulated 1.3 ± 0.5, 1.8 ± 0.2, and 1.8 ± 0.2 times at p30, respectively (Figure 3A).

To verify that the expression of let-7e and let-7i was in Müller cells, immunofluorescence staining was performed against GS together with fluorescence *in situ* hybridization for let-7e and let-7i. We found that let-7e and let-7i co-localized with GS in the somas and processes of Müller cells of RCS-p+ rats. The intensity of let-7e and let-7i signals in RCS-p+ rat retinas was stronger than that of controls at early stages of retinal degeneration, p15 and p30 (Figure 3B–3C3). These results suggested that in RCS-p+ rat retinas the levels of let-7e and let-7i increased in Müller cells, which may diminish Müller cell de-differentiation and proliferation during retinal degeneration.

### Downregulation of Lin28B may upregulate let-7 family molecules

We tested the expression level of Lin28B using Western blotting since previous studies have shown that the developmentally regulated RNA-binding protein, Lin28, selectively repressed the expression of let-7 microRNA [36]. We found that Lin28B expression only increased before retinal degeneration at p1 and p7 in RCS-p+ rat retinas compared with controls. The expression of Lin28B declined in RCS-p+ rat retinas at the beginning of retinal degeneration after rats opened their eyes at p15 and was significantly decreased with progressive degeneration at p30, p60, and p90 (Figure 3D and 3D1). These data suggested that reduced expression of Lin28B may increase expression of the let-7 family in Müller cells from RCS-p+ rat retinas.

### Ectopic Lin28B expression promotes the stem cell phenotype of Müller cells *in vitro*

**Figure 4:**
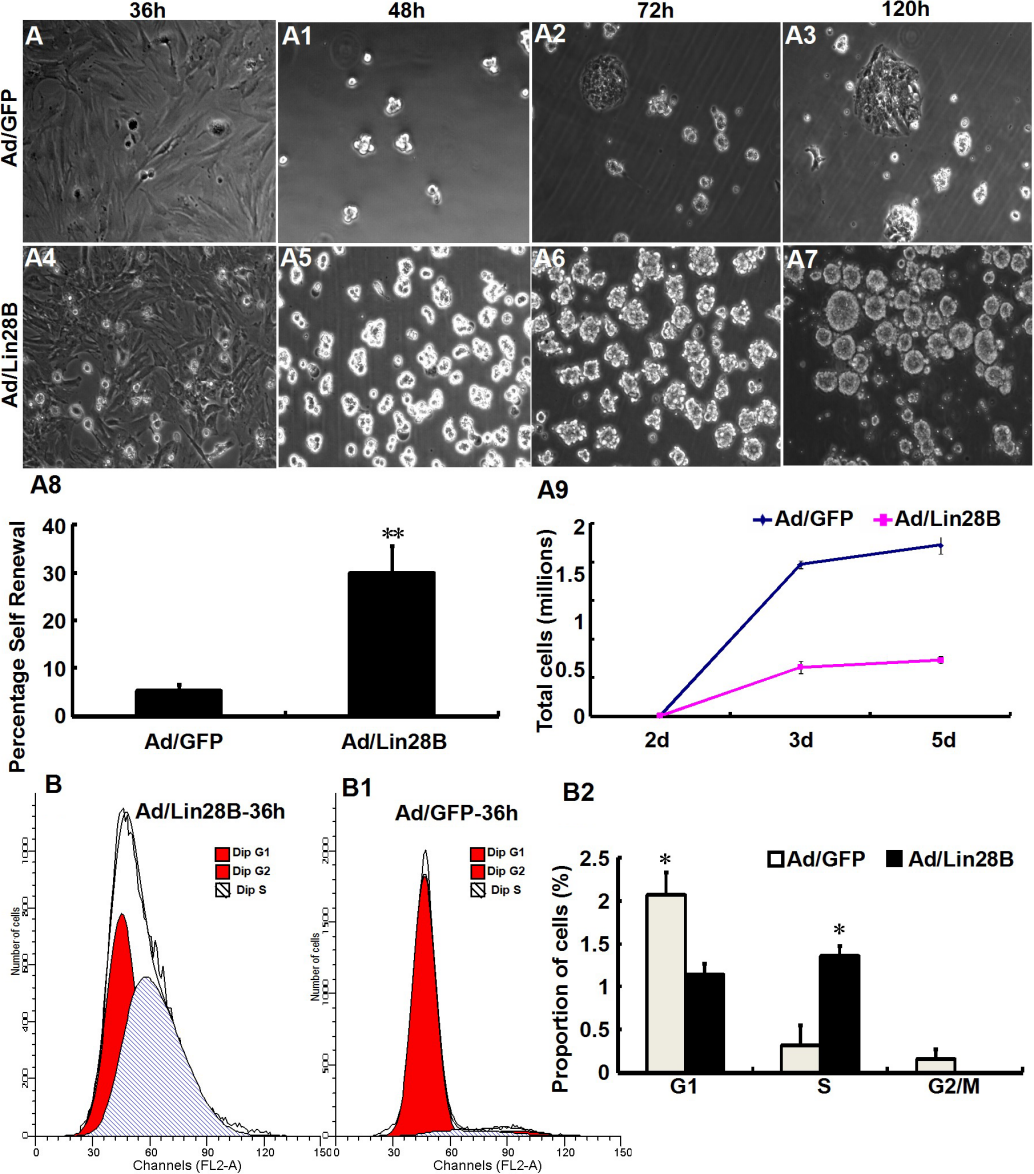
Ectopic Lin28B expression promotes the stem cell phenotype and inhibits glial phenotype of Müller cells *in vitro* (**A**–**A9**) Clone formation efficiency of cultured Müller cells treated with Ad/Lin28B was higher than that treated with Ad/GFP. Both self-renewal ability (A8) and proliferation (A9) of Ad/Lin28B infected group were significantly stronger compared with the Ad/GFP infected group. (**B**–**B2**) DNA content analysis of Müller cells treated with Ad/Lin28B or Ad/GFP by flow cytometry. There was a significant increase in the percentage of S phase in Müller cells treated with Ad/Lin 28B compared with those treated with Ad/GFP. Representative results are shown. Data are presented as the mean ± standard error from three replicates. *P < 0.05, **P < 0.01, Student's *t*-test.

We tested self-renewal in Müller cells cultures infected with Ad/Lin28B and Ad/GFP. Müller cells infected with Ad/Lin28B had short, thick processes and proliferated extensively compared with Müller cells infected with Ad/GFP. The infected cells dissociated into single cells after 36 h and were plated in stem cell medium at a clonal density of 1 cell/μl. The number of secondary spheres that formed were counted after 2, 3, and 5 days. Cultured Müller cells treated with Ad/Lin28B began to quickly divide in stem cell medium 48 h after passaging and expanded clonogenically. They formed small, uneven peripheral spheres 72 h after passaging, then, they formed larger round and uneven-edged spheres 120 h after infection (Figure 4A4–4A7). Cultured Müller cells treated with Ad/GFP were more likely to grow adherently, exhibited an uneven periphery with loose intercellular adhesion (Figure 4A–4A3). Approximately 30 ± 6% of Müller cells infected with Ad/Lin28B gave rise to secondary spheres, indicating that the spheres contained self-renewing stem cells. By contrast, only 5 ± 1% of Müller cells infected with Ad/GFP formed secondary spheres (Figure 4A8). The cell population doubling rates were calculated 2, 3, and 5 days after plating cells in stem cell medium. The doubling time of Müller cells infected with Ad/Lin28B was 0.091 h, more than 3-fold quicker than cells infected with Ad/GFP (0.285 h) (Figure 4A9).

Cell-cycle analysis of cultured Müller cells 36 h after infection with Ad/Lin28B or Ad/GFP was carried out by FACS. We found that the percentage of S phase cells in Ad/Lin28B treated Müller cells was significantly higher than that of cells treated with Ad/GFP (54 ± 5% vs. 13 ± 9%), and the percentage of G1 phase cells in Ad/Lin28B treated Müller cells was significantly lower than cells treated with Ad/GFP (46 ± 5% vs. 83 ± 11%) (Figure 4B–4B2).

### Ectopic Lin28B expression decreases the accumulation of let-7 family miRNAs and promotes Müller cells de-differentiation *in vivo*

**Figure 5:**
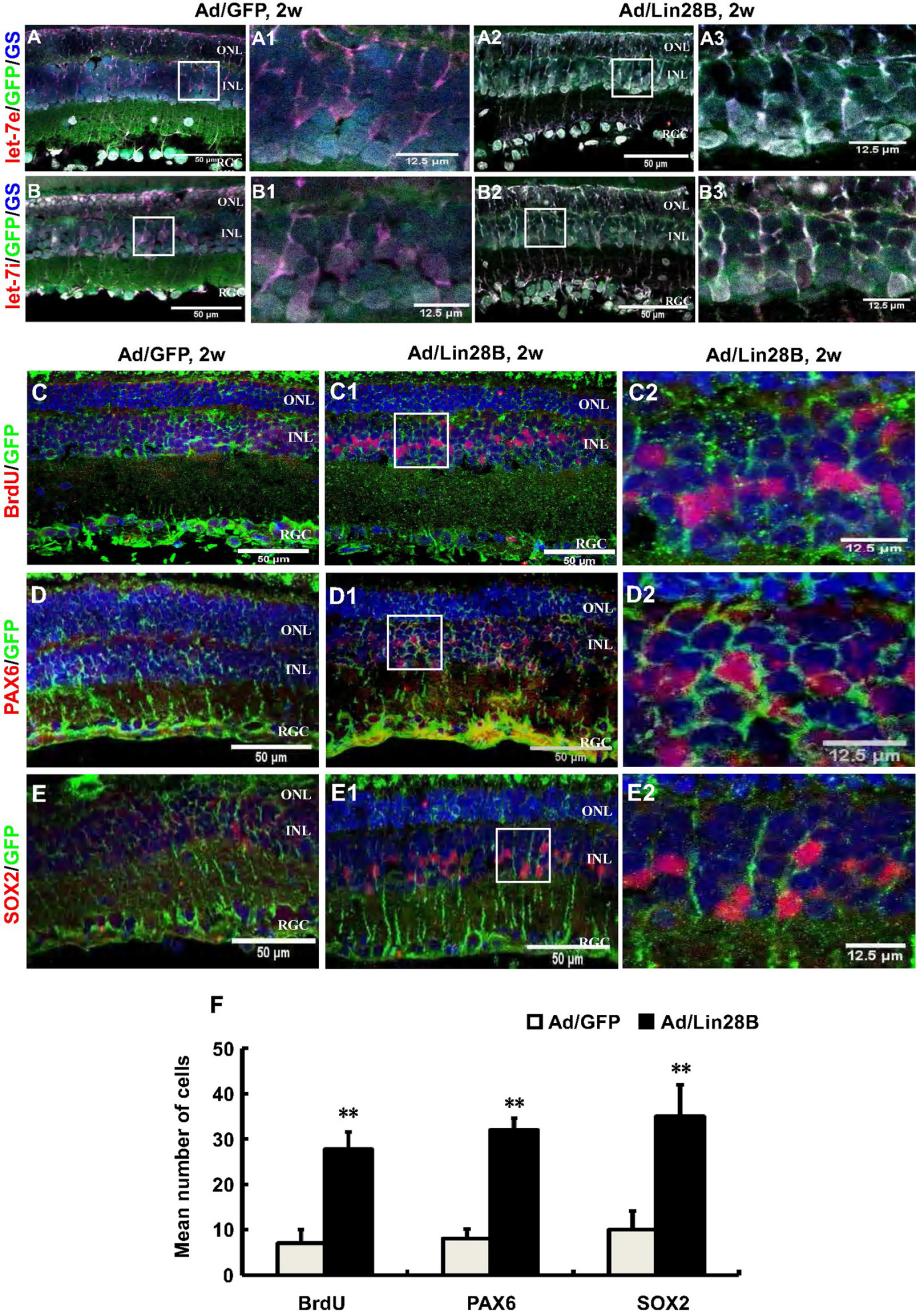
Ectopic Lin28B expression decreases the accumulation of let-7 family miRNAs and promotes Müller cells de-differentiation *in vivo* (**A**–**B3**) Immunofluorescence staining against GS (blue) and *in situ* hybridization with LNA let-7e or let-7i probes (red) at 2 weeks after subretinal space injection of Ad/Lin28B or Ad/GFP (green) in RCS-p+ rats. The color of blue nuclei stained with DAPI was transformed to white because a blue fluorophore was used to label GS, a Müller cell marker. A1, B1 and A3, B3 are enlargements of the square in (A, B and A2, B2). The results showed reduced expressions of let-7e and let-7i in the Ad/Lin28B infected group compared with Ad/GFP infected group. (**C**–**E2**) Immunofluorescence staining against the cell proliferation marker BrdU and progenitor markers PAX6 and SOX2 (red) at 2 weeks after subretinal space injection of Ad/Lin 28B or Ad/GFP (green) in RCS-p+ rats. C2–E2 are enlargements of the square in C1–E1. (**F**) Quantitative and statistical analysis showed that the number of positive cells of BrdU, PAX6 and SOX2 in the Ad/Lin28B infected group were significantly increased when compared with Ad/GFP infected group. Representative results are shown. Data are presented as the mean ± standard error from three replicates. *P < 0.05, **P < 0.01, Student's *t*-test.

To further verify that Lin28B expression down-regulation in dystrophic retinas causes let-7 family molecule accumulation, we used an adenoviral expression system to overexpress Lin28B in the retina by delivering Ad/Lin28B into the subretinal space of RCS-p+ rats at p21. The Ad/GFP was used as a control. Immunofluorescence staining against GS combined with fluorescent *in situ* hybridization for let-7e or let-7i at 2 weeks after Ad/Lin28B or Ad/GFP administration was performed. We found that the intensity of let-7e and let-7i signals in Müller cells were significantly down-regulated in Ad/Lin28B treated retinas when compared with the Ad/GFP treated retinas (Figure 5A–5B3).

We confirmed that the ectopic expression of Lin28 *in vivo* promoted the stem cell phenotype of Müller cells by delivering Ad/Lin28B into the subretinal space of RCS-p+ rats. The rats that received Ad/Lin28B or Ad/GFP were injected intraperitoneally with BrdU (50 mg/kg) 2 h before sacrificing. Expression of proliferative marker BrdU and stem/progenitor cell markers PAX6 and SOX2 were studied by immunofluorescence staining and cell counting at 2 weeks after delivery. We found that there were more PAX6 and SOX2 positive cells that double stained with GFP in the INL of retinas treated with Ad/Lin28B than retinas treated with Ad/GFP (Figure 5C–5F). These results suggested that ectopic Lin28B expression specifically promotes the stem cell phenotype of Müller cells *in vivo*.

### Ectopic Lin28B expression promotes neurogenesis and inhibits gliogenesis of de-differentiated Müller cells *in vivo*

**Figure 6:**
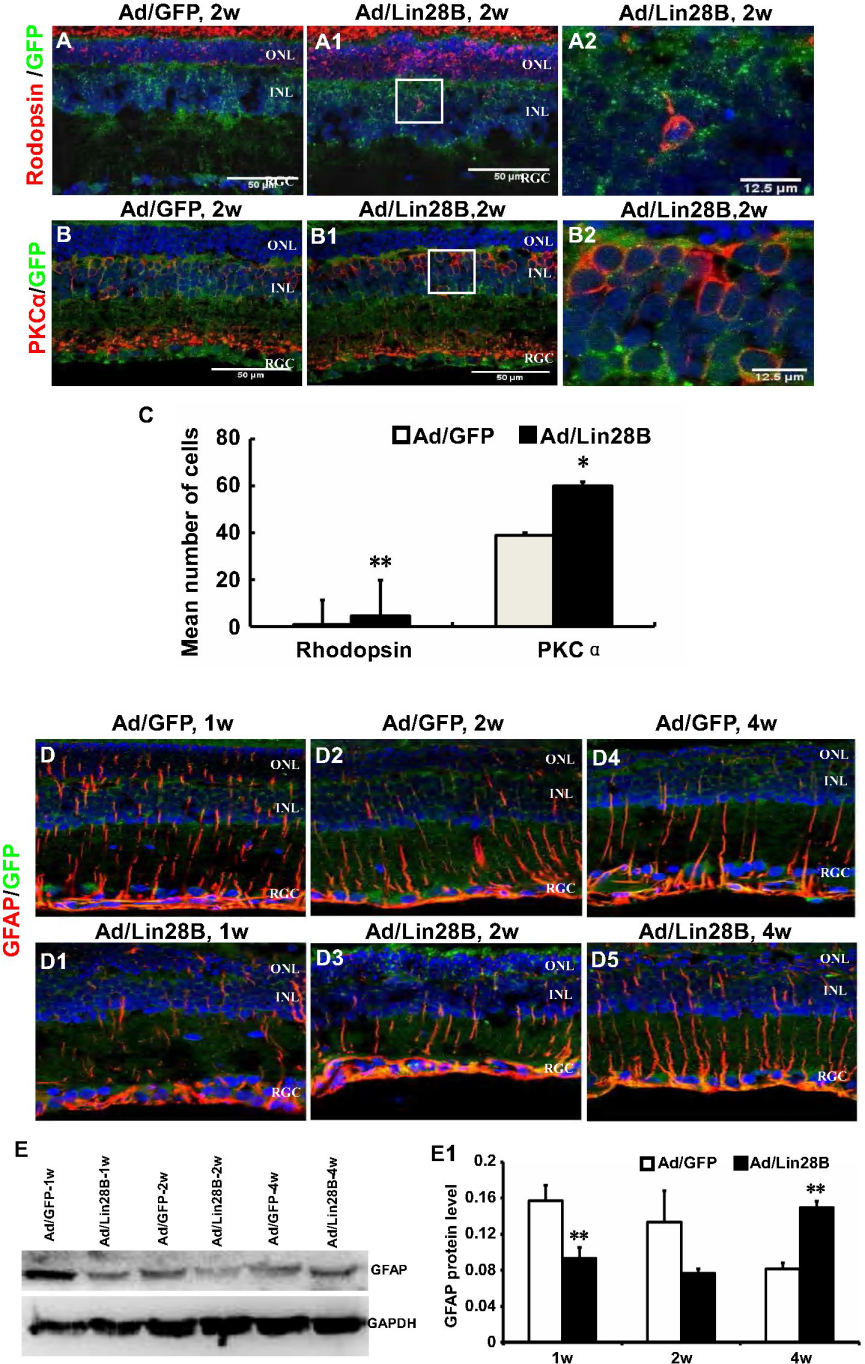
Ectopic Lin28B expression promotes neurogenesis and inhibits gliogenesis of de-differentiated Müller cells *in vivo* (**A**–**B2**) Immunofluorescence staining against retinal neural markers rhodopsin and PKCα at 2 week after subretinal space injection of Ad/Lin 28B or Ad/GFP (green) in RCS-p+ rats. A2 and B2 are enlargements of the square in of A1–B1. (**C**) Quantitative and statistical analysis showed that the positive cells of rhodopsin and PKCα in the Ad/Lin28B infected group were significantly increased when compared with the Ad/GFP infected group. (**D**–**D5**) Immunofluorescence staining against GFAP at 1 week, 2 weeks, and 4 weeks after subretinal space injection of Ad/Lin 28B or Ad/GFP (green) in RCS-p+ rats. The result showed that the level of GFAP expression gradually decreased in Ad/Lin 28B treated retinas as compared to the retinas treated with Ad/GFP. (**E**–**E1**) Western blot analysis supported the result of immunofluorescence staining. Representative results are shown. Data are presented as the mean ± standard error from three replicates. *P < 0.05, **P < 0.01, Student's *t*-test.

We tested whether de-differentiated Müller cells in the retinas treated with Ad/Lin28B *in vivo* could also trans-dedifferentiate into neurons, including photoreceptors in RCS-p+ rats. Immunofluorescence staining was performed on retinas treated with Ad/Lin28B or Ad/GFP with the antibodies for rhodopsin (labels rod photoreceptor outer segments) and PKC α (specific for retinal rod bipolar cells). We found that the retinas infected with Ad/Lin28B expressed more rhodopsin in the ONL and also a few rhodopsin/GFP positive cells were found in the INL (Figure 6A–6A2, 6C). The number of PKCα/GFP and calbindin/GFP positive cells were both increased in the INL after Ad/Lin28B infection compared with control retinas (Figure 6B–6B2, 6C). We also observed the expression of GFAP and found it gradually decreased with Lin28B overexpression in RCS-p+ rat retinas, while in the retinas of the control group a high level of GFAP expression was maintained (Figure 6D–6D5). This was confirmed with Western blotting (Figure 6E and 6E1). These data suggested that Müller cells expressing Ad/Lin28B lost their gliosis characteristics and had the capacity to trans-differentiate towards other types of retinal neurons after de-differentiation.

### Upregulating Lin28B expression may rescue visual function

**Figure 7:**
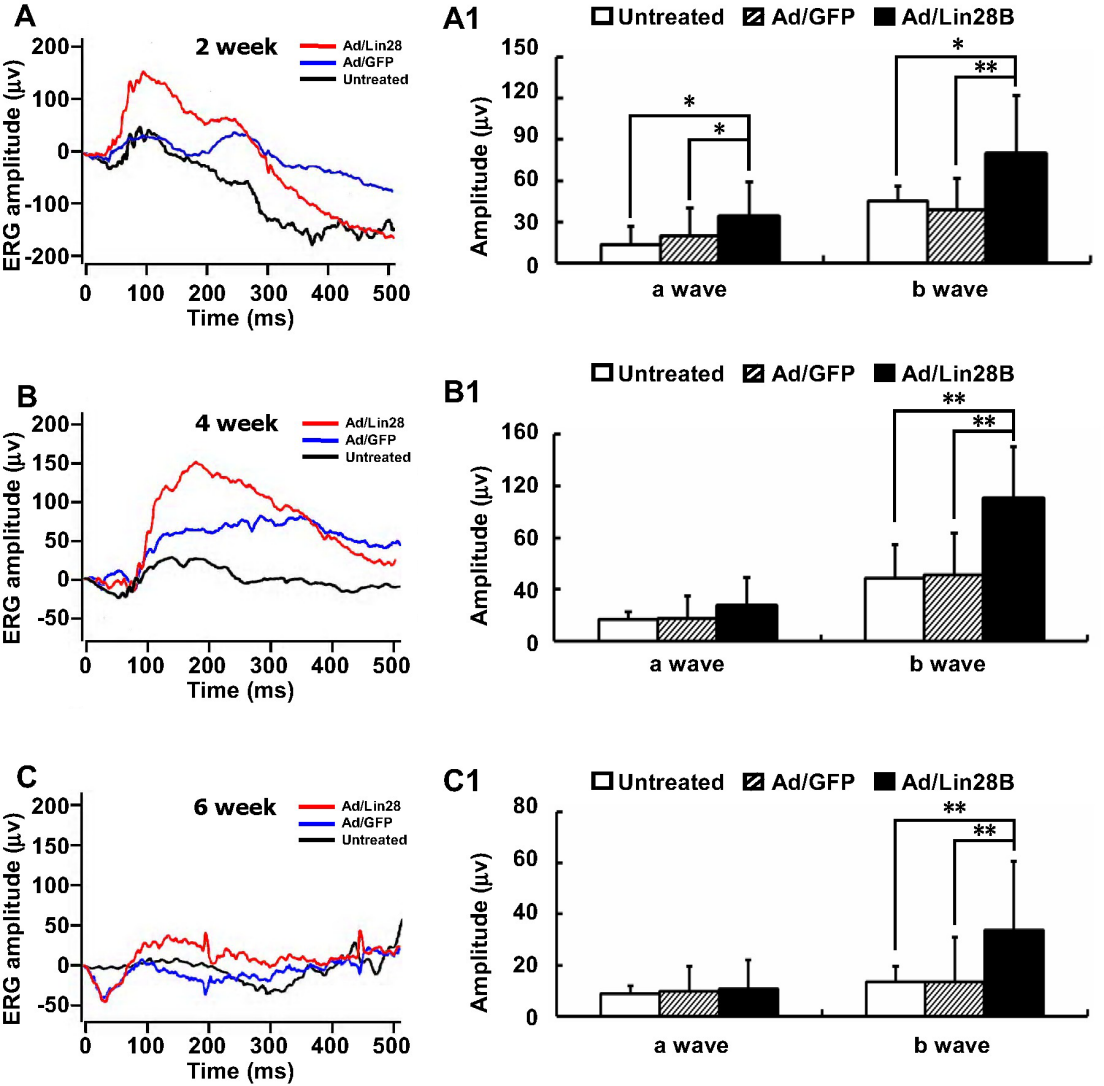
Ectopic Lin28B expression may restore visual function (**A**–**C1**) ERG recordings (0dB) of RCS-p+ rats' eyes in untreated group (black line), Ad/Lin28B (red line) or Ad/GFP (blue line) injection groups. Both a-wave and b-wave amplitudes of Ad/Lin28B treated eyes were significantly greater than untreated eyes and Ad/GFP treated eyes at 2 weeks (A, A1) (*n* = 8). The increased b-wave amplitudes reached a peak at 4 weeks (B, B1) (*n* = 5) after Ad/Lin28 treatment and remained significantly greater at 6 weeks (C, C1) (*n* = 5) when compared with the untreated eyes and Ad/GFP treated eyes. Representative results are shown. Data are presented as the mean ± standard error from three replicates. *P < 0.05, **P < 0.01, Student's *t*-test.

We measured the visual function of RCS-p+ rats untreated or treated with Ad/Lin28B or Ad/GFP with flash ERG. Robust responses were detected in 18 rats in response to scotopic stimuli to eyes infected with Ad/Lin28B, while the ERG responses of 28 rats untreated or receiving Ad/GFP were depressed to the same level as dystrophic control rats, demonstrating that neither the injection procedure nor the presence of non-functional adenovirus resulted in false positive responses. The efficacy rate of Ad/Lin28B was about 64.3%, while no effective vision improvement was observed in untreated or Ad/GFP treated eyes. Compared with untreated and Ad/GFP treated eyes, the mean a-wave amplitude tended to increase from 13 ± 13 μv and 20 ± 20 μv to 34 ± 26 μv (*n* = 8) 2 weeks after treatment with Ad/Lin28B and the mean b-wave amplitude increased significantly from 45 ± 42 μv and 39 ± 23 μv to 79 ± 43 μv (*n* = 8) (Figure 7A and 7A1). Four weeks after infecting the retinas with Ad/Lin28B, the mean a-wave amplitude increased from 17 ± 5 μv and 17 ± 18 μv to 27 ± 22 μv (*n* = 5), and the mean b-wave amplitude increased from 48 ± 26 μv and 51 ± 33 μv to 110 ± 40 μv (*n* = 5) (Figure 7B and 7B1). Six weeks after delivering Ad/Lin28B, although the amplitudes were less than that of 4 weeks, the a-wave amplitudes of control eyes and treated eyes were 8.9 ± 9.9 μv, 9.7 ± 9.9 μv and 11 ±12 μv, respectively, and the b-wave amplitudes were significantly different, 13 ± 17 μv, 14 ± 17 μv and 34 ± 27 μv (*n* = 5), respectively (Figure 7C and 7C1). There were no significant differences in the a-wave latency and b-wave latency among the three groups at all time points. These data demonstrated that Ad/Lin28B improved visual function in dystrophic RCS rat retinas (Student's *t*-test, *P* < 0.01 in all cases).

## DISCUSSION

We observed that Müller cells had a transient de-differentiation and proliferation in the retina of RCS-p+ rats and identified that Lin28B, an evolutionarily conserved RNA-binding protein, may stimulate Müller cell de-differentiation by down-regulating the expression of the microRNAs let-7e and let-7i. This down regulation promoted the Müller cells to trans-differentiate into a variety of retinal cell types. Many studies indicated that glia in the CNS of adults can be reprogrammed into neural stem cells or pregenitors and participate in neural regeneration [37]. For instance, astrocytes and NG2-glia (oligodendrocyte precursors) may be stimulated as endogenous CNS cells after brain injury [38, 39]. It has shown that Müller cells in rat retinas can acquire a progenitor phenotype in chronic retinal disease or when treated with neurotrophic factors or during exogenous stem cell transplantation [13, 40–43]. In this study, we found that at the early stages of retinal degeneration, Müller cells expressed the proliferative marker BrdU, stem/progenitor markers PAX6 and SOX2. However, Müller cells quickly become glial scar with the development of retinal degeneration, as the microenvironment of degenerative retinas might be not suitable for the survival of retinal progenitors [3, 44].

Considering the growing evidence implicates that miRNAs have a variety of effects on glia including development, differentiation, activation [45], as well as cell fate determination [46, 47], we performed microRNA array analysis of RCS rat retinas and found that let-7 miRNA family molecules seemed to have a strikingly different expression pattern in RCS-p+ rats compared with control rats. The expression of most let-7 miRNA family molecules was increased, especially let-7e and let-7i, which were upregulated 4 times and 12 times, respectively in dystrophic rat retinas. The major functions of the let-7 family include regulation of cell cycle progression and cell proliferation. The prominent genes regulated by let-7 consist of those involved in executing cell-fate decisions, oncogenes and cell-cycle factors [47]. Let-7 deletions are likely to stimulate the cell cycle and DNA synthesis [27]. Recently, it revealed that the let-7 family regulated posttranscriptional genetic circuits involved in the heterochronic pathway that regulated developmental timing and aging in *C. elegans* [48]. The intrinsic timing mechanism that controls the developmental decline in neuronal regeneration depends on the progressive increase of let-7 in neurons [49]. In teleost fish, let-7a and let-7f miRNA expression was dramatically reduced with injury in Müller cell derived progenitors so that the injured retinas were able to regenerate and be completely repaired by de-differentiated Müller cells [26].

Our results suggested that overexpression of let-7e and let-7i might be the specific event that blocks de-differentiation and further proliferation of Müller cells in degenerated retinas. We explored why let-7e and let-7i were upregulated in Müller cells during retinal degeneration. Recent studies have found that Lin28, miRNA-binding proteins, directly block the biogenesis of let-7 miRNAs post-transcriptionally by binding to the terminal loop region of the let-7 primary or precursor miRNAs (pri- or pre-miRNAs) of miRNAs in mammalian cells [36, 50–53]. In present study, we found the level of Lin28B was reduced in RCS-p+ rat retinas compared with control rats. We believed that it might be Lin28B that negatively regulated the levels of let-7e and let-7i. Our results are in accordance with previous studies on zebrafish which demonstrated that Lin28 and let-7 were involved in the de-differentiation of Müller cells after retina injury [26].

We proposed that upregulating Lin28B might repress overexpression of let-7e and let-7i in the Müller cells during retinal degeneration, thereby tightly controlled their de-differentiation and proliferation. To verify this assumption, we used an adenoviral vector to upregulate the expression of Lin28B in Müller cells of RCS-p+ rats, while Ad/GFP was used as control. We confirmed that the expression levels of let-7e and let-7i miRNAs were significantly repressed in Müller cells transfected Ad/Lin28B, as the cells gained features of stem/progenitor cells. Infected Müller cells expressed BrdU, PAX6 and Sox2 signals more strongly, formed more colonies and had a greater capacity to self-renew than Müller cells infected with Ad/GFP. We also observed that the transfected Müller cells had the potential to generate lamina-specific retinal neurons. They expressed rhodopsin and PKC α proteins, while the expression of GFAP was suppressed. These results suggested that expression of Lin28B might promote Müller cells to acquire a neural fate and to inhibit the glial fate.

We found that the visual function of 64% of the eyes treated with Ad/Lin28B were partially restored compared with untreated or Ad/GFP treated eyes. There were no significant differences in the ERG responses between the three groups 1 week after Ad/Lin28 administration. In fact, there was a decreasing trend in a-wave and b-wave amplitudes in the group treated with Ad/Lin28 compared with untreated or Ad/GFP treated group. This might be resulted from the de-differentiation of Müller cells at that point, which caused the loss of many functions of Müller cell. However, 2 weeks after Ad/Lin28 administration, both a-wave and b-wave amplitudes of eyes treated with Ad/Lin28 were significantly greater than those of untreated or Ad/GFP treated group and the b-wave amplitudes peaked at 4 weeks and remained significantly greater than that of controls at 6 weeks.

ERG provides a gross measure of light mediated trans-retinal function, averaged across the whole retina [54]. Thus, we speculated that the possible reasons for increased ERG a- or b- wave amplitudes were come from the de-differentiation of Müller cells which gradually acquired a neural fate and established a robust integration with second order neurons, and formed efficient visual functional units. Another possibility was that the de-differentiated Müller cells reduced glial scar formation and improved the homeostasis of degenerative retina. However, the elevated amplitudes of a- and b-waves dropped again at 4 weeks and 6 weeks, after Lin28B administration respectively, it perhaps was a result of developing retinal degeneration.

In summary, we proposed that the activation of Müller cells produced a transitory progenitor state that quickly resulted in gliogenesis under the regulation of let-7e and let-7i in the degenerative retina of RCS-p+ rats. We found that upregulating Lin28B RNA binding protein induced Müller cells to remain in a pluripotent state by repressing let-7e and let-7i expression, which promoted de-differentiated Müller cells to acquire a neuronal fate. Our findings underlined the importance of the opposite effects between Lin28B and let-7e and let-7i on Müller cells, which played an important role in promoting retinal regeneration and repair. These data provide a point for further studies to uncover the underlying mechanisms of the Lin28B and let-7 signaling pathway mediated de-differentiation and proliferation of Müller cells with the ultimate aim of developing endogenous stem cells for regeneration and repair of retinal degenerative diseases.

## MATERIALS AND METHODS

### Animals and ethics statement

RCS-rdy^+^-p^+^ rats (of either sex) were used as controls for the test RCS-p^+^ rats. RCS rats at postnatal day 15 (p15), p30, p45, p60, and p90, and age-matched rdy^+^ controls were housed in a temperature-controlled room on a 12-hour light–dark cycle in the Laboratory Animal Unit of Third Military Medical University. All the experimental and animal handling procedures complied with the Association for Research in Vision and Ophthalmology Statement and were also reviewed and approved by the Faculty Committee on the Use of Live Animals in Teaching and Research, Third Military Medical University.

### Primary Müller cell culture

Briefly, eyes from postnatal days 8–10 of control rats were enucleated and incubated for 6–8 h in Dulbecco's Modified Eagle Medium (DMEM). Eyecups were transferred to dissociation solution DMEM containing 0.1% Trypsin, 0.02% EDTA and 70 U/ml collagenase, and incubated at 37°C in a CO_2_ incubator for 1 h. Eyecups were washed with DMEM containing 10% fetal bovine serum (FBS) and 1% antibiotic-antimycotic mixture, and the retinas were dissected with care to avoid contamination of RPE and ciliary epithelium. The retinas were mechanically dissociated into small aggregates and cultured in DMEM containing 10% FBS and 1% glutamax. After 7 days, the floating retinal aggregates and debris were removed leaving Müller cells attached to the bottom of the dish. Cells were trypsinized and cultured in DMEM containing 10% FBS for another 5 days to obtain a 2-fold purified population of cells. Once these cultures attained confluence, the cells were trypsinized and used for immunocytochemistry.

### Cell cycle analysis

Samples were collected over indicated time points and fixed in 70% ethanol overnight. For cell cycle analysis, fixed cells were treated with RNase for 20 min before the addition of 5 μg/mL propidium iodide and analyzed by FACS.

### Western blot

For the detection of the protein expression levels of PAX6, SOX2, Lin28B, and GFAP, the RCS and control rat retinas in each group were homogenized in ice-cold radioimunoprecipitation assay lysis buffer (50 mM of Tris-HCl buffer (pH 7.4), 150 mM NaCl, 1% Triton X-100, 1% sodium deoxycholate, 0.1% SDS, sodium orthovanadate, sodium fluoride, EDTA, and leupeptin). The homogenate was then centrifuged at 12,000 × g for 5 min at 4°C. The clear supernatants were stored at −80°C until use. Protein concentration was determined using a bicinchoninic acid Kit (Beyotime Institute of Biotechnology, China). The samples (50 μg of protein/lane) were loaded and electrophoresed on a 12% sodium dodecylsulfate-polyacrylamide gel for 40 min at 120 V. The proteins were transferred from the gel onto a nitrocellulose (NC) membrane for 70 min at 120 V. After transferring, the NC membranes were blocked with block solution containing Tris-buffered saline, 0.1% Tween 20 (TBST) and 5% free-fat milk for 1 h at room temperature. The blots were then washed three times (5 min each time) with TBST, and then incubated with primary antibodies (Shown in Table 1) overnight at 4°C. After washing, all membranes were again incubated with GAPDHor actin antibody for 1 h at room temperature. Afterwards, all membranes were incubated in different secondary antibodies each for 1 h at room temperature while shaking. Between incubations, the membranes were rinsed. Finally, the NC membranes were scanned using an Odyssey infrared imaging system with the Odyssey Application software V1.2.15 for PAX6, SOX2, Lin28B and GFAP bands. The relative levels of PAX6, SOX2, Lin28B and GFAP were obtained by calculating the ratio of PAX6, SOX2, Lin28B and GFAP to GAPDH or actin, respectively.

**Table 1:** Antibodies used in immunofluorescence staining, Western blot and ISH

Antibody	Company	Con.	Speci	Cat#	Purpose
PAX6	Santa Cruz Biotechnology	1:200/1:1000	Rabbit	sc-11357	IF/WB
SOX2	Abcam	1:300/1:1000	Rabbit	ab97959	IF/WB
Lin28B	Cell Signaling	1:1000	Mouse	5422S	WB
GS	Millipore	1:600	Mouse	Mab302	
GFAP	Abcam	1:800/1:1000	Rabbit	ab48050-100	IF/WB
Rhodopsin	Abcam	1:300	Mouse	ab81702	IF
PKC-α	Abcam	1:500	Mouse	Ab32376	IF
Brdu	Cell Signaling	1:100	Mouse	5292S	IF
Anti-DIG-AP	Roche	1:300	Sheep	REF11093274910	ISH
GAPDH	Cwbio, China	1:2000	rabbit	CW0101	Internal control
β-actin	Cwbio, China	1:1000	mouse	CW0096	Internal control
Secondary antibodies Cy3 (mouse)	Beyotime, China	1:500	goat	A0521	IF
Secondary antibodies Cy3 (rabbit)	Beyotime, China	1:500	goat	A0516	IF
Secondary antibodies Cy3 (goat)	Beyotime, China	1:500	donkey	A0502	IF
Secondary antibodies Cy3 (mouse)	GeneTex	1:500	donkey	GTX85338	IF
Secondary antibodies FITC (sheep)	Santa Cruz Biotechnology	1:50	donkey	SC-2467	ISH
Secondary antibodies FITC (goat)	Santa Cruz Biotechnology	1:200	donkey	SC-2024	IF
Secondary antibodies FITC (mouse)	Zhongshan goldenridge, China	1:200	goat	ZF-0312	IF
Secondary antibodies Alexa-647 (sheep)	Jackson	1:100	donkey	713-605-003	IF
Secondary antibodies Alexa-647 (mouse)	Beyotime, China	1:500	goat	A0473	IF
Secondary antibodies Alexa-647 (Rabbit)	Beyotime, China	1:500	goat	A0468	IF

### Immunofluorescence staining

For immunofluorescence staining of frozen sections, control and experimental rats were anesthetized with tribromoethanol (1 mg/kg; Aldrich) and perfused with 4% paraformaldehyde (PFA) in phosphate buffer saline (PBS). Retinas were isolated by dissecting the eyes from their orbits, then lightly brushing the retina apart from the sclera and pigment epithelium. They were then rinsed in 0.01 M phosphate buffer. Isolated eyes were further fixed with 4% PFA/PBS for 1 h prior to incubation in 30% sucrose/PBS for 16 h followed by freezing in opti-mum cutting temperature compound (OCT) cryopreservation medium. Frozen sections (10 μm) of OCT-embedded eyes were made and then stored at −80°C. The sections were air dried, washed in PBS for 5 min, and blocked with 3% bovine serum albumin and were thereafter incubated with a primary rabbit-anti body for PAX6, SOX2, BrdU, GFAP, rhodopsin and PKCα, respectively in 0.25% Triton-X-100 (Sigma-Aldrich, USA) and 0.25% bovine serum albumin (Sigma-Aldrich, USA) in PBS overnight at 4°C (Shown in Table 1). The sections were then washed three times (5 min per wash) and incubated with the secondary antibody for 1 h at room temperature (all secondary antibodies were shown in Table 1). All sections were mounted after washing and the nuclei were counterstained with 4′,6-diamidino-2-phenylindole.

### *In situ* hybridization

MirRCURY LNA Let-7e and Let-7i RNA detection probes, rno-Let-7e: CUAUACGGCCUCCUAGCUUUCC (Prod. No. 202888) and rno-Let-7i: UGAGGUAGU AGUUUGUGCUGUU (Prod. No. 202234), were labeled with 5′- and 3′-digoxigenin and provided by EXIQON (Vedbaek, Denmark). Retinal sections from both the RCS-P+ and control rat retinas were processed in parallel and incubated overnight at 60°C with either antisense or sense probes. The hybridization signal was detected using the anti-digoxigenin antibody, 5-bromo-4-chloroindolyl-phosphatase and nitroblue tetrazolium (Roche Applied Biosciences, Basel, Switzerland).

### Delivery of Adeno-Lin28B

Lin28B Adenovirus was made with an AdMax™ system (SBO Medical Biotechnology, Shanghai, China). The transgene was cloned into shuttle plasmids which was then co-transfected into 293 cells with an Ad genomic plasmid (pBHG frt ΔE1,3 FLP). High efficiency site specific recombination catalyzed by FLP recombinase resulted in “rescue” of the expression cassette into the left end of an E1 deleted (first generation) Ad vector. The Adeno-Lin28B was harvested from 293T cells. Meanwhile, adenovirus with EGFP was delivered to the opposite eye, used as a negative control. The integration unit was 1.05E+09 per ml. Adeno-Lin28B (5 μl) was administered into the subretinal space to infect retinal cells of RCS-p+ rats at p20 days. After 1, 2, and 4 weeks of injection, the eyes were enucleated and frozen sections of the samples were analyzed by western blot analysis.

### ERG recording

Animals were dark adapted for nearly 12 h and prepared for recording under dim red light. Anesthesia was induced with an intramuscular thigh injection of amine (100 mg/kg) and xylazine (12 mg/kg). Pupils were dilated with one drop each of tropicamide and phenylephrine. Corneal ERG responses were recorded from both eyes simultaneously with gold wire loops. A drop of 0.9% saline was frequently applied onto the cornea to prevent its dehydration and to allow electrical contact with the recording electrode. Two of the needle electrodes were inserted under the scleral conjunctiva around the equator of the eyes and served as the reference electrodes. The other electrode was placed in the tail and served as the ground. Amplification (at 0.1–500Hz band pass, without notch filtering), stimulus presentation, and data acquisition were provided by the Reti-scan system (Roland, Germany). Dark-adapted intensity responses ranged from 0.0003 to 9.5cd.s.m-2 (0.0003, 0.03, 0. 3, 0.95, 3.0 and 9.5cd.s.m-2). To avoid any adapting effect of the previous flash, the inter-flash interval was 10–120s depending on the stimulus intensity.

### Images capture and analysis

Images were taken using a digital camera mounted on a microscope after immunofluorescence staining.

### Statistics

Unless otherwise stated, 5 animals were used in each group. All eyes were used to count the number of positive cells. The number of positive cells was counted in 20 areas of each section. All data were presented as mean and standard error of the mean. Data were evaluated using a non-parametric statistical method or Student's *t*-test statistical analysis for comparing means at the same time point between the experiment and control group. Values of *P* < 0.05 were considered to be significant.
